# Uptake and Acceptability of Information and Communication Technology in a Community-Based Cohort of People Who Inject Drugs: Implications for Mobile Health Interventions

**DOI:** 10.2196/mhealth.3437

**Published:** 2015-06-25

**Authors:** Andrew Genz, Gregory Kirk, Damani Piggott, Shruti H Mehta, Beth S Linas, Ryan P Westergaard

**Affiliations:** ^1^ Department of Epidemiology Johns Hopkins Bloomberg School of Public Health Baltimore, MD United States; ^2^ Department of Medicine Johns Hopkins University School of Medicine Baltimore, MD United States; ^3^ Department of Medicine University of Wisconsin-Madison Madison, WI United States

**Keywords:** substance abuse, intravenous, Internet, cellular phone, text messaging, telemedicine

## Abstract

**Background:**

Mobile phone and Internet-based technologies are increasingly used to disseminate health information and facilitate delivery of medical care. While these strategies hold promise for reducing barriers to care for medically-underserved populations, their acceptability among marginalized populations such as people who inject drugs is not well-understood.

**Objective:**

To understand patterns of mobile phone ownership, Internet use and willingness to receive health information via mobile devices among people who inject drugs.

**Methods:**

We surveyed current and former drug injectors participating in a longitudinal cohort study in Baltimore, Maryland, USA. Respondents completed a 12-item, interviewer-administered questionnaire during a regular semi-annual study visit that assessed their use of mobile technology and preferred modalities of receiving health information. Using data from the parent study, we used logistic regression to evaluate associations among participants’ demographic and clinical characteristics and their mobile phone and Internet use.

**Results:**

The survey was completed by 845 individuals, who had a median age of 51 years. The sample was 89% African-American, 65% male, and 33% HIV-positive. Participants were generally of low education and income levels. Fewer than half of respondents (40%) indicated they had ever used the Internet. Mobile phones were used by 86% of respondents. Among mobile phone owners, 46% had used their phone for text messaging and 25% had accessed the Internet on their phone. A minority of respondents (42%) indicated they would be interested in receiving health information via phone or Internet. Of those receptive to receiving health information, a mobile phone call was the most favored modality (66%) followed by text messaging (58%) and Internet (51%).

**Conclusions:**

Utilization of information and communication technology among this cohort of people who inject drugs was reported at a lower level than what has been estimated for the general U.S. population. Our findings identify a potential barrier to successful implementation of mobile health and Internet-based interventions for people who inject drugs, particularly those who are older and have lower levels of income and educational attainment. As mobile communication technology continues to expand, future studies should re-examine whether mHealth applications become more accessible and accepted by socioeconomically disadvantaged groups.

## Introduction

Utilization of mobile phones for communication and access to information has become nearly ubiquitous in both low- and high-income countries. At the end of 2013, the number of active mobile phone subscriptions worldwide was estimated at 6.8 billion, or approximately 96 subscriptions for every 100 inhabitants of the world [1]. The global availability of mobile broadband services is rapidly increasing. In the foreseeable future, the majority of the world’s population will have access to the Web via a mobile device. With this expanded access to increasingly powerful handheld devices has come recognition of potential applications for improving health and health care [2].

Persons affected by substance abuse and poverty face substantial challenges in consistently accessing and utilizing health care for chronic medical conditions. Successful chronic disease management typically requires consistent health-promoting behaviors such as daily medication administration, periodic monitoring of disease-specific biomarkers, and clinical assessments by medical providers. Maladaptive, drug-seeking behaviors and material deprivation that are frequently associated with substance use disorders may interfere with all of these steps. For example, people who inject drugs (PWID) have been shown to have inferior medication adherence and more frequent interruptions in therapy when treated for human immunodeficiency virus (HIV) [3,4], hepatitis C virus (HCV)[5,6], and tuberculosis[7,8]. PWID also have high rates of depression [9-11] and alcohol dependence [11,12], chronic disorders for which regular clinical monitoring is important for preventing disability and poor social functioning. In view of these challenges, strategies that leverage information and communication technology (ICT) to enhance care delivery and support treatment adherence among PWID could play an important role in reducing health disparities.

Growth in the number of health-related apps and steady reduction in costs for mobile devices has created the potential for mobile communication technology to add considerable value to medical care and public health programs, particularly among populations with limited resources. Mobile health, or mHealth, has been broadly defined as medical or public health practice supported by mobile devices. If demonstrated to be effective, affordable and scalable, mHealth approaches could be uniquely beneficial to medically underserved or marginalized patient groups, who may encounter geographic or socioeconomic barriers to health care or have limited access to timely and relevant health information.

If mobile health apps tailored to the needs of PWID are demonstrated to be feasible and efficacious, additional translational research will be necessary in order to understand whether and how they can be successfully implemented in clinic and community settings. A recent cross-sectional survey of 100 consecutive HIV-infected patients seen at an urban clinic in Baltimore, Maryland demonstrated near-universal mobile phone ownership (96%), and a high level of willingness to use mobile phones for medication adherence support [13]. However, relatively few respondents owned a smartphone (28%) or had used a mobile phone to view the Web content (34%). These proportions are somewhat lower than estimates that 51% of Americans owned a smartphone in 2012 [14].

The goal of the present study was to gain insight into the real-world feasibility and acceptance of mHealth approaches among a cohort of older, predominantly African American PWID in a largely poor, urban community in the United States. We conducted a cross-sectional survey among volunteers in a long-running community-based cohort study in order to evaluate mobile phone ownership and use of the Web for health-related purposes. Based on the research team’s experience and personal interaction with the study participants and the high median age of the active cohort (51.8 years), we hypothesized that the adoption of ICT among our study sample would be lower than the levels reported in previous studies.

## Methods

### Recruitment

The study sample was comprised of participants in the AIDS Linked to the Intravenous Experience (ALIVE) cohort who were active in the study during 2011. ALIVE is a community-based cohort study that has continuously followed current and former PWID in Baltimore since 1988 [15]. The population has a high prevalence of HIV infection, viral hepatitis, and substance use disorders. Previous research with this cohort has demonstrated suboptimal levels of treatment utilization [16,17] and high levels of multimorbid chronic conditions [18,19], characteristics that make this group an attractive target for mHealth-based enhancements to routine clinical care.

### Data Collection

Between March and September of 2011, all ALIVE participants attending a regularly scheduled, semiannual study assessment were invited to complete an additional 12-item, interviewer-administered questionnaire. The questionnaire was developed by investigators specifically for this sub-study, and contained items assessing ownership of mobile phones, and utilization of phones for voice calls, texting or data download. Individuals’ lifetime history of Web access and patterns of recent Web use were assessed with multiple choice questions.

Study participants who volunteered to complete the supplemental ICT questionnaire were notified that the ALIVE investigators were exploring different ways to collect and disseminate health information among PWID. In this context, all participants were asked whether they would be willing to receive health information via voice calls on a mobile phone, text messages on a mobile phone, or using the Internet. The full text of the questionnaire is available in Multimedia Appendix 1.

Baseline sociodemographic characteristics (e.g., race, sex, age, education) were taken from participants’ baseline assessments conducted at enrollment in the parent study. For the subset of respondents who were HIV-positive, clinical parameters reflecting effective treatment (e.g., CD4+ cell count, HIV viral load, use of antiretroviral therapy) were captured from the most recent ALIVE study visit.

### Statistical Analysis

For the present analysis, the two questionnaire items of foremost concern were “Do you currently own a cell phone?” and “Have you ever used the Internet?” Responses to these items comprised the primary outcome variables. We compared the frequency of “yes” responses to these items across categories of sociodemographic and clinical characteristics. Chi-squared tests were used to assess whether any individual characteristics were associated with phone ownership and Web use, assuming that a p-value less than or equal to 0.05 indicated a statistically significant bivariate association.

Using logistic regression, we calculated adjusted odds ratios to estimate the independent association between individual characteristics and the two main outcomes. Covariates included in the model were age, race/ethnicity, gender, education level and amount of legal income in the past six months. These variables were selected based on prior research suggesting that patterns of Web and mobile phone use vary across categories of these demographic characteristics. All analyses were performed using STATA version 11. The Institutional Review Board at the Johns Hopkins Bloomberg School of Public Health reviewed and approved the study protocol.

## Results

###  Sample Characteristics

Of 1,024 individuals invited to complete the questionnaire between March 7, 2011 and September 29, 2011, 845 agreed to participate, yielding a response rate of 82.5% (Table 1). The median age of participants was 51.8 years (IQR=47.0-56.7). Of these, 89.2% were African American, 65.1% were male, and 40.6% had a high school diploma or GED. A majority of respondents (55.9%) reported that their legal income during the prior six months was less than $5000.

Approximately one third of respondents (275/845, 32.5%) were known to be HIV seropositive at the time of the survey, and 84.0% (710/845) were seropositive for hepatitis C virus. Of the HIV-infected subgroup, 76.0% (209/275) were receiving antiretroviral therapy and 50.2% (138/275) of these had an undetectable HIV viral load, representing fully effective HIV treatment.

**Table 1:** Participant characteristics (N=845).

Characteristics	n (%)^a^
Age (median, IQR)^b^	51.8 (46.9-56.6)
Female	295 (34.9)
Male	550 (65.1)
African American	754 (89.2)
Finished high school or GED	342 (40.6)
Legal income during past 6 months	
None	157 (18.6)
$0 - $4,999	473 (55.9)
$5,000 or higher	215 (25.4)
Homeless in past 6 months	58 (6.9)
Current smoker	660 (78.2)
Alcohol use in past 6 months	407 (47.2)
Injected drugs in past 6 months	207 (24.5)
HIV positive	275 (32.5)
HCV^c^ positive	710 (84.0)
HIV viral load undetectable^d^	138 (50.4)
CD4+ cell count (median, IQR)^d^	408 (255-659)
Currently taking ART^e^	209 (76.0)

^a^ All values presented are N(%) unless otherwise specified

^b^ IQR=interquartile range

^c^ HCV=hepatitis C virus

^d^ Clinical variables presented only for 275 HIV-infected respondents

^e^ ART=antiretroviral therapy

### Mobile Phone and Web Use

Responses to questionnaire items assessing mobile phone ownership, use of phones for texting and Web use are summarized in Table 2. Of the 845 respondents asked, 86.0% (727) reported owning a mobile phone at the time of the survey. Over half of these (56.7%, 412/727) subscribed to a monthly payment plan through a wireless carrier; 25.7% (187/727) utilized a prepaid or pay-as-you-go payment system; 45.9% (334/727) used a free phone provided by a government program. Most respondents (64.9%, 549/845) reported having a single phone number (including mobile and land lines) during the three months preceding the survey, but 9.9% (84/845) used three or more numbers and 0.8% (7/845) used 10 or more numbers during that time. Nearly all respondents (92.2%, 779/845) had heard of free government phone programs (e.g. Safelink or “Obamaphone”) and about half (50.1%, 423/845) had utilized such a program. See Multimedia Appendix 2 for these details on participants’ phone use practices.

Four participants had incomplete information on both phone use questions.

Approximately half of participants (46.2%, 334/723) who owned a mobile phone reported sending or receiving text messages. Fewer (18.5%, 134/723) reported they use a mobile phone to access the Web. Lifetime Web use was low in this cohort. Overall, 40.5% (342/845) reported ever using the Web. Table 3 presents a detailed account of responses to these questions by demographic characteristics and HIV infection status.

**Table 2:** Participant responses to key questions.

	Owns a mobile phone (n=845)^a^	Uses a mobile phone for texting (n=723)^b^	Uses a mobile phone for Web (n=723)^b^	Ever used Web (n=845)^a^
	n (%)	n (%)	n (%)	n (%)
Overall	727 (86.0)	334 (46.2)	134( 18.5)	342 (40.5)

^a^ N=all 845 participants surveyed

^b^ n=only the 723 participants who owned a mobile phone

**Table 3:** Breakdown of mobile phone ownership, text messaging, and Web use by demographic characteristics and HIV status.

		Owns a mobile phone (n=845)	Uses a mobile phone for texting (n=723)	Uses a mobile phone for Web (n=723)	Ever used Web (n=845)
		n (%)	n (%)	n (%)	n (%)
**Gender**					
	Male	463 (84.2)	193 (41.9)	79 (17.2)	228 (41.5)
	Female	264 (89.5)	141(53.8)	55 (20.9)	114 (38.6)
**Race**					
	Non-AA	73 (80.2)	39 (53.4)	23 (31.5)	58 (63.7)
	AA	654 (86.7)	295 (45.4)	111 (17.1)	284 (37.7)
**Age**					
	<40	63 (88.7)	42 (66.7)	23 (36.5)	46 (64.8)
	41-50	222 (84.1)	118 (53.1)	54 (24.3)	126 (47.8)
	51-60	353 (86.3)	141 (40.4)	45 (12.9)	136 (33.3)
	60+	89 (86.0)	33 (37.1)	12 (13.5)	34 (33.7)
**Education**					
	No HS/GED	435 (87.0)	182 (42.0)	70 (16.2)	165 (33.0)
	HS/GED	289 (84.5)	151 (52.6)	64 (22.2)	175 (51.2)
**Legal income** ^b^					
	$0	126 (80.3)	45 (35.7)	14 (11.1)	44 (28.0)
	<$5000	409 (86.5)	195 (47.8)	76 (18.6)	184 (38.9)
	>$5000	192 (89.0)	94 (49.7)	44 (23.3)	114 (53.0)
**HIV status**					
	HIV-negative	487 (85.4)	218 (44.9)	93 (19.1)	240 (42.1)
	HIV-positive	240 (87.0)	116 (49.0)	41 (17.3)	102 (37.1)
^a^ Percent is for each row, for each question. (e.g. 84.2% of men had a phone and 15.8% of men did not)					
^b^ During six months prior to questionnaire					

There were numerous disparities in mobile phone and Web use across demographic and socioeconomic strata. Mobile phone ownership was more prevalent among women, African Americans, and those with higher income (Table 4). Reporting any Web use was independently associated with younger age, completion of high school and higher income (Table 5). African American respondents were half as likely to report ever using the Web, an association that remained statistically significant after adjusting for age, education and income (adjusted OR 0.5, 95% CI 0.3 – 0.9).

Among the 310 Web-using respondents, there were no differences by race or gender in the proportion who reported accessing the Web using a mobile phone. Based on the multivariate model, the only factor associated with using phones to access the Web was age. Respondents who were under 50 were more than twice as likely to report mobile Web use as those who were older than 50.

**Table 4:** Associations among mobile phone use and selected characteristics.

		Owns mobile phone (n=842)		Uses phone for text messaging (n=720)	
		Adjusted OR^a^	95% CI ^a^	Adjusted OR ^a^	95% CI ^a^
**Gender**					
	Male	reference		reference	
	Female	1.6	1.0 – 2.5	1.5	1.1 – 2.1
**Race**					
	Non-African American	reference		reference	
	African American	1.8	1.0 – 3.4	1.2	0.7 – 2.1
**Age**					
	<40	reference		reference	
	41-50	0.5	0.2 – 1.2	0.4	0.2 – 0.9
	51-60	0.6	0.2 – 1.4	0.3	0.1 – 0.5
	60+	0.7	0.2 – 1.9	0.2	0.1 – 0.5
**Education**					
	Less than HS/GED	reference		reference	
	Completed HS/GED	0.8	0.5 – 1.2	1.6	1.2 – 2.3
**Legal income, past 6 months**					
	$0	reference		reference	
	<$5000	1.5	1.0 – 2.5	1.7	1.1 – 2.6
	>$5000	2.1	1.1 – 3.8	2.0	1.2 – 3.2
**HIV status**					
	HIV-negative	reference		reference	
	HIV-positive	1.1	0.7 – 1.7	1.2	0.9 – 1.7
**Taking ART** ^b^					
	Yes	reference		reference	
	No	1.1	0.5 – 2.7	0.7	0.4 – 1.3
**HIV viral load** ^b^					
	Undetectable	reference		reference	
	Detectable	0.4	0.2 – 1.0	0.8	0.4 – 1.4

^a^ Adjusted for gender, race, age, educational level, and income

^b^ Model limited to 275 HIV-positive participants

**Table 5:** Associations among Web use and selected characteristics.

		Ever used Web (n=842)		Accessed Web using mobile phone (n=310)	
		Adjusted OR^a^	95% CI ^a^	Adjusted OR ^a^	95% CI ^a^
**Gender**					
	Male	reference		reference	
	Female	0.9	0.6 – 1.2	1.4	0.9 – 2.3
**Race**					
	Non-African American	reference		reference	
	African American	0.5	0.3 – 0.9	1.1	0.5 – 2.2
**Age**					
	<40	reference		reference	
	41-50	0.6	0.3 – 1.0	0.7	0.3 – 1.6
	51-60	0.3	0.1 – 0.5	0.4	0.2 – 0.9
	60+	0.2	0.1 – 0.5	0.4	0.2 – 1.5
**Education**					
	Less than HS/GED	reference		reference	
	Completed HS/GED	2.1	1.5 – 2.8	1.0	0.6 – 1.5
**Legal income, past 6 months**					
	$0	reference		reference	
	<$5000	1.8	1.2 – 2.8	1.7	0.8 – 3.8
	>$5000	3.4	2.1 – 5.5	1.8	0.7 – 4.1
**HIV status**					
	HIV-negative	reference		reference	
	HIV-positive	0.8	0.6 – 1.1	0.9	0.6 – 1.5
**Taking ART** ^b^					
	Yes	reference		reference	
	No	0.5	0.2 – 0.9	0.6	0.2 – 1.4
**HIV viral load** ^b^					
	Undetectable	reference			
	Detectable	0.4	0.2 – 0.8	0.6	0.3 – 1.3

^a^ Adjusted for gender, race, age, educational level, and income

^b^ Model limited to 275 HIV-positive participants

### Responses Among HIV-Infected PWID

HIV-positive and HIV-negative respondents had similar levels of mobile phone ownership and Web use. Among the HIV-positive cohort, 87.3% of participants (240/275) reported owning a mobile phone, and this proportion did not vary by disease stage or self-reported use of antiretroviral therapy. However, compared with HIV-positive respondents who successfully achieved viral suppression, respondents who had a detectable HIV viral load at the most recent ALIVE visit reported lower levels of ICT adoption. Fewer participants with detectable HIV viremia owned mobile phones (83.0%, 224/270 vs. 90.6%, 125/138) and used the Web (31.9%, 86/270 vs. 42.8%, 59/138) than their virally suppressed counterparts. After adjusting for gender, race, age, education and income, PWID with uncontrolled HIV infection were significantly less likely to own a mobile phone (adjusted OR 0.4, 95% CI 0.2 – 1.0) and to use the Web (adjusted OR 0.4, 95% CI 0.2 – 0.8).

### Willingness to Receive Health Information Via Mobile Phone and Web

**Figure 1:**
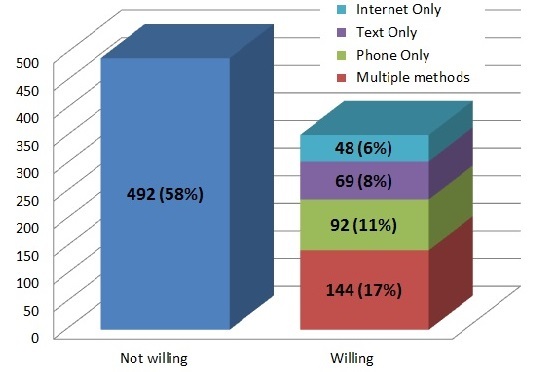
Willingness to receive health information via mobile phone, text message or internet (N=845).

The majority of respondents (58.2%, 492/845) indicated they would not like to receive health information via mobile phone, text message, or Web. Of the 353 respondents who expressed willingness to receive health information via one or more ICT modalities, 65.7% (232/353) indicated they would like to receive health information by phone, 57.5% (203/353) were willing to receive health-related text messages, and 50.7% (179/353) would use the Web to receive health information (Figure 1). When queried about the type of health information they would be willing to receive, 62.3% (220/353) indicated they would be interested in information about smoking cessation and 58.4% (206/353) would utilize ICT for medication reminders. Most indicated they would prefer health-related communications to be infrequent. Only 15.6% (55/353) preferred daily communication, while larger proportions favored weekly (25.5%, 90/353) or monthly (42.2%, 149/353) communication.

## Discussion

### Principal Findings

In this cross-sectional study of former and current PWID, we observed a high prevalence of mobile phone ownership, but lower-than-expected levels of Web use. Relatively few PWID in this cohort expressed interest in using information and communication technology for monitoring or improving health. Taken together, these findings suggest that at the time of the survey, PWID in Baltimore may not be highly receptive to health promotion interventions featuring mobile phones or Web-based communication. As of late 2011, about 60% of the ALIVE cohort reported they had never used the Web, and only about 1 in 5 considered the Web a desirable means of receiving health information.

Whether these results should temper enthusiasm about mHealth approaches to chronic disease management among PWID depends on several factors. The ALIVE cohort consists of low income, inner city residents who are generally poorly educated and therefore have limited employment opportunities. Over half (58%; 631/1088) of all ALIVE participants seen in 2011 had not graduated from high school. It is possible that members of this cohort will be “late adopters” of mobile technology because they have been economically disadvantaged, but will become increasingly receptive to mHealth strategies as mobile devices and data subscriptions become more accessible and affordable. Our results could alternatively reflect structural barriers based on local economic factors and service availability, or even more deeply seated resistance to adopting technology related to cultural norms. Future surveys to monitor changes in technology use among this population will help clarify whether the limited uptake of ICT persists.

### Comparison With Prior Work

Prior surveys performed among similar urban-dwelling African American individuals agreed with our findings that mobile phone ownership and use are commonplace. There is evidence, however, that despite widespread use of mobile phones, the use of mobile technology for communicating health information has not been adopted equally among subgroups with older age and less education. A recent survey among women attending a sexually transmitted diseases clinic in Baltimore showed high levels of mobile phone ownership (93%), text messaging (79%), and Web use (80%) [20]. Among women in this study, those who were older and had lower levels of education were significantly less willing to receive health information via mobile phone. A survey among ethnic minority parents attending an urban pediatric clinic similarly showed near-universal mobile phone ownership, but found that only 17% of respondents ever shared or received health information via text messaging [21].

While adoption of ICT may be lagging in the communities comprising our study population, there is growing evidence that mobile devices and Web-based apps are feasible and acceptable to PWID in research settings. For example, heroin- and cocaine-dependent patients have effectively used handheld devices to monitor real-time experiences of stress, drug cravings, and drug use [22,23]. As previously described by our research group, mobile phone-based ecologic momentary assessment (EMA) methods are also being evaluated for real-time collection of data relevant to both drug use and antiretroviral treatment adherence [24].

Our study found significant disparities in ICT adoption by race. African American respondents were more likely to own a mobile phone than white or Hispanic participants, but were substantially less likely to have used the Web. Low-income, educationally disadvantaged minority communities have been considered a “digitally underserved” population based on previous surveys [25]. Whether race or ethnicity is independently predictive of low ICT uptake is less clear. Contrary to our findings, a 2012 report published by the National Urban League found evidence that African Americans used broadband Internet to apply for jobs more often than white Americans, and that this discrepancy was most pronounced among those without a high school diploma and an annual income of less than $20,000 [26].

To our knowledge, the finding of an association between Web use and successful HIV treatment has not been previously reported. Among HIV-infected respondents in our study, those who received ART and achieved an undetectable HIV viral load had more than twice the odds of reporting they used the Web. While this association is not likely causal in nature, it suggests that patients at the highest risk for suboptimal treatment outcomes are least likely to be current adopters of ICT. This represents a potentially important barrier to implementing mHealth solutions among patients who stand to benefit from them most. Treatment of HIV infection exemplifies the complex requirements for successful long-term disease management. Without high levels of medication adherence, viral replication may continue unchecked, leading to immune system dysfunction, elevated risk of antiretroviral drug resistance, and higher likelihood of HIV transmission. If mHealth modalities are to have a potentially beneficial role in facilitating treatment engagement for high-risk patients, implementation strategies may need to address the same social and structural barriers that tend to limit the effectiveness of medical care.

### Limitations

The usual limitations of cross-sectional research apply to our results. The landscapes of mobile technology and patterns of Web use are continually changing. In this study sample with relatively low levels of ICT adoption, it is to be expected that individuals’ use of technology has evolved since the time the data were collected. Because this survey was conducted among a socioeconomically homogenous sample of PWID in a single U.S. city, our findings cannot likely be generalized to many other contexts. Related strengths of the study are its large sample size and moderately high response rate among a population that is not well represented in previous research on this topic.

### Conclusions

As behavioral determinants of health play a central role in chronic disease management for patients who use drugs, there may be uniquely beneficial applications of mHealth technology for supporting the special needs and vulnerabilities of drug-using patients. Moreover, the capacity for mobile data collection and processing, combined with a growing marketplace for software apps has fostered the development of innovative and sophisticated approaches to monitoring symptoms and even promoting and facilitating behavior change. Successful implementation of these concepts outside the research setting will require an accurate understanding of the adoption of ICT among targeted populations. Strategies to eliminate barriers to ICT utilization may be essential components of mHealth interventions aimed at improving health among PWID and other marginalized groups.

## Appendix

Multimedia Appendix 1 The ALIVE ICT questionnaire.

Multimedia Appendix 2 Frequencies of mobile phone ownership among ALIVE participants.

## Abbreviations

ALIVE: AIDS linked to the intravenous experience
EMA: ecologic momentary assessment
HIV: human immunodeficiency virus
ICT: information and communication technology
PWID: people who inject drugs

## References

[1] (2013). International Telecommunication Union (ITU).

[2] Collins F (2012). How to fulfill the true promise of “mHealth”. Sci Am.

[3] Mugavero MJ, Lin HY, Allison JJ, Giordano TP, Willig JH, Raper JL, Wray NP, Cole SR, Schumacher JE, Davies S, Saag MS (2009). Racial disparities in HIV virologic failure: do missed visits matter?. J Acquir Immune Defic Syndr.

[4] Yehia BR, Fleishman JA, Metlay JP, Moore RD, Gebo KA (2012). Sustained viral suppression in HIV-infected patients receiving antiretroviral therapy. JAMA.

[5] Mehta Shruti H, Lucas Gregory M, Mirel Lisa B, Torbenson Michael, Higgins Yvonne, Moore Richard D, Thomas David L, Sulkowski Mark S (2006). Limited effectiveness of antiviral treatment for hepatitis C in an urban HIV clinic. AIDS.

[6] Mehta SH, Genberg BL, Astemborski J, Kavasery R, Kirk GD, Vlahov D, Strathdee SA, Thomas DL (2008). Limited uptake of hepatitis C treatment among injection drug users. J Community Health.

[7] Horsburgh CR, Goldberg S, Bethel J, Chen S, Colson PW, Hirsch-Moverman Y, Hughes S, Shrestha-Kuwahara R, Sterling TR, Wall K, Weinfurter P, Tuberculosis Epidemiologic Studies Consortium (2010). Latent TB infection treatment acceptance and completion in the United States and Canada. Chest.

[8] Caylà JA, Rodrigo T, Ruiz-Manzano J, Caminero JA, Vidal R, García JM, Blanquer R, Casals M, Working Group on Completion of Tuberculosis Treatment in Spain (Study ECUTTE) (2009). Tuberculosis treatment adherence and fatality in Spain. Respir Res.

[9] Springer SA (2012). High rates of depressive symptomatology among injecting drug users in Saskatoon, Canada. Evid Based Ment Health.

[10] Pilowsky DJ, Wu LT, Burchett B, Blazer DG, Ling W (2011). Depressive symptoms, substance use, and HIV-related high-risk behaviors among opioid-dependent individuals: results from the Clinical Trials Network. Subst Use Misuse.

[11] Mackesy-Amiti ME, Donenberg GR, Ouellet LJ (2012). Prevalence of psychiatric disorders among young injection drug users. Drug Alcohol Depend.

[12] Kidorf M, Disney ER, King VL, Neufeld K, Beilenson PL, Brooner RK (2004). Prevalence of psychiatric and substance use disorders in opioid abusers in a community syringe exchange program. Drug Alcohol Depend.

[13] Miller CWT, Himelhoch S (2013). Acceptability of Mobile Phone Technology for Medication Adherence Interventions among HIV-Positive Patients at an Urban Clinic. AIDS Res Treat.

[14] Duggan M (2012). Rainie, Cell Phone Activities.

[15] Vlahov D, Anthony JC, Munoz A, Margolick J, Nelson KE, Celentano DD, Solomon L, Polk BF (1991). The ALIVE study, a longitudinal study of HIV-1 infection in intravenous drug users: description of methods and characteristics of participants. NIDA Res Monogr.

[16] Kavasery R, Galai N, Astemborski J, Lucas GM, Celentano DD, Kirk GD, Mehta SH (2009). Nonstructured treatment interruptions among injection drug users in Baltimore, MD. J Acquir Immune Defic Syndr.

[17] Westergaard RP, Hess T, Astemborski J, Mehta SH, Kirk GD (2013). Longitudinal changes in engagement in care and viral suppression for HIV-infected injection drug users. AIDS.

[18] Piggott DA, Muzaale AD, Mehta SH, Brown TT, Patel KV, Leng SX, Kirk GD (2013). Frailty, HIV infection, and mortality in an aging cohort of injection drug users. PLoS One.

[19] Salter ML, Lau B, Go VF, Mehta SH, Kirk GD (2011). HIV infection, immune suppression, and uncontrolled viremia are associated with increased multimorbidity among aging injection drug users. Clin Infect Dis.

[20] Samal L, Hutton HE, Erbelding EJ, Brandon ES, Finkelstein J, Chander G (2010). Digital divide: variation in internet and cellular phone use among women attending an urban sexually transmitted infections clinic. J Urban Health.

[21] Mitchell S, Godoy L, Shabazz K, Horn IB (2014). Internet and mobile technology use among urban African American parents: survey study of a clinical population. J Med Internet Res.

[22] Epstein DH, Willner-Reid J, Vahabzadeh M, Mezghanni M, Lin JL, Preston KL (2009). Real-time electronic diary reports of cue exposure and mood in the hours before cocaine and heroin craving and use. Arch Gen Psychiatry.

[23] Preston KL, Epstein DH (2011). Stress in the daily lives of cocaine and heroin users: relationship to mood, craving, relapse triggers, and cocaine use. Psychopharmacology (Berl).

[24] Kirk G, Linas BS, Westergaard RP, Piggott D, Bollinger RC, Chang LW, Genz A (2013). The exposure assessment in current time study: implementation, feasibility, and acceptability of real-time data collection in a community cohort of illicit drug users. AIDS Res Treat.

[25] Bates K, Malakoff L, Kane S, Pulidindi J (2012). National Leage of Cities.

[26] Wijewardena M, Hardy V (2012). National Urban League Policy Institute.

