# Supplementary material for: Uptake and Acceptability of Information and Communication Technology in a Community-Based Cohort of People Who Inject Drugs: Implications for Mobile Health Interventions
Source: JMIR Mhealth Uhealth. 2015 Jun 25;3(2):e70. doi: 10.2196/mhealth.3437 (PMC4526964; doi:10.2196/mhealth.3437)
Supplement: Multimedia Appendix 1 [file mhealth_v3i2e70_app1.pdf]

THE ALIVE STUDY

ICT QUESTIONNAIRE

READ TO THE PARTICIPANT:

This questionnaire is about your cell phone and internet use. We are looking at new ways to collect information from you. Some of the ideas we are looking at include using cell phones and the internet. This information can help us improve this study and other new studies. Please be completely honest, your responses will not affect your participation in the ALIVE Study or any other study.

SECTION A

CELL PHONE USE

READ TO THE PARTICIPANT:

This first section asks questions about your cell phone use.

A1. Do you have a cell phone right now?

NO	0	**(A4)**
YES	1	

A2. What type of payment plan do you have?

		NO	YES	Refused	Don't Know
a	**Monthly Plan** (e.g. AT&T, Verizon)	0	1	7	8
b	**Pay-as-you-go** (e.g. Cricket, Boost)	0	1	7	8
c	**Other (specify):** ______________ ______________________________	0	1	7	8
	**CODE** ____ ____				

A3. In addition to making calls, do you use your phone for **(Choose all that apply)**:

		NO	YES	Refused	Don't Know
a	**Send/Receive Text Messages**	0	1	7	8
b	**Internet**	0	1	7	8
c	**Other (specify):** ______________ ______________________________	0	1	7	8
	**CODE** ____ ____				

A4. In the past 3 months, how many different cell phone numbers have you used? ____

A5. Have you ever heard of **Safe Link** or **Assurance**, government programs that provide free cell phone service to people on government assistance? ***NOTE: Some might call it the "Obamaphone".**

NO	0	**(B1)**
YES	1	

A6. Did you or anyone you live with get a phone from **Safe Link** or **Assurance**?

NO	0
YES	1

SECTION B

INTERNET USE

READ TO THE PARTICIPANT:
This section asks questions about your internet use.

B1. Have you ever used the internet?

NEVER	0	**(C1)**
YES	1	

B2. How often do you use the internet?

EVERY DAY	1
FEW DAYS PER WEEK	2
FEW DAYS PER MONTH	3
RARELY	4

B3. Where do you use it? **(Choose all that apply)**

		NO	YES	Refused	Don't Know
**a**	**Computer at your home**	0	1	7	8
**b**	**Computer at friend or family home**	0	1	7	8
**c**	**On your cell phone**	0	1	7	8
**d**	**Library**	0	1	7	8
**e**	**Internet café or business**	0	1	7	8
**f**	**Other (specify): _______________ ________________________________**	0	1	7	8
	**CODE ____ ____**				

SECTION C

ACCEPTABILITY

READ TO THE PARTICIPANT:

The ALIVE Study might try some new ideas in future studies.

Please tell us what you think about the following ideas, **even if you do not have a computer or cell phone.**

***NOTE: Text/calls/internet would be free of charge for study purposes!!!**

C1. Would you like to receive health advice by? **(Choose all that apply)**

		NO	YES	Refused	Don't Know
a	**Internet**	0	1	7	8
b	**Cell phone call**	0	1	7	8
c	**Text message**	0	1	7	8

*** If they <u>never</u> reply "YES" for C1, then skip to END (7) & enter time.**

C2. How often would you like to receive health advice (by phone or internet)?

EVERY DAY	1
FEW DAYS PER WEEK	2
FEW DAYS PER MONTH	3
RARELY	4

C3. Which type of health advice would you like to receive (by phone or internet)? **(Choose all that apply)**

		NO	YES	Refused	Don't Know
a	**Medication reminders**	0	1	7	8
b	**Stopping or reducing drug use**	0	1	7	8
c	**Stopping or reducing smoking**	0	1	7	8
d	**Stopping or reducing drinking**	0	1	7	8
e	**Other (specify): _______________ ________________________________**	0	1	7	8
	**CODE ____ ____**				
