# Supplementary material for: Uptake and Acceptability of Information and Communication Technology in a Community-Based Cohort of People Who Inject Drugs: Implications for Mobile Health Interventions
Source: JMIR Mhealth Uhealth. 2015 Jun 25;3(2):e70. doi: 10.2196/mhealth.3437 (PMC4526964; doi:10.2196/mhealth.3437)
Supplement: Multimedia Appendix 2 [file mhealth_v3i2e70_app2.pdf]

Frequencies of Mobile Phone Ownership

Among ALIVE Participants (N=845)

	n (%)
Do you have a cell phone right now?	
Yes	727 (86.0)
What type of cell phone payment plan do you have?	
Monthly	412 (48.8)
Pay-as-you-go	128 (15.1)
Free	187 (22.1)
No phone plan	118 (14.0)
In the past 3 months, how many different phone numbers have you had?	
0	77 (9.1)
1	549 (65.0)
2	122 (14.4)
3-4	69 (8.2)
5-9	10 (1.2)
10+	7 (0.8)
Not sure/Didn't answer	11 (1.3)

Have you ever heard of any free government phone program? (e.g. Safelink or “Obamaphone”)	
Yes	779 (92.2)
Did you or anyone you live with get a phone from a government program?	
Yes	423 (50.1)
